# Asymptomatic filariasis and leprosy co-morbidity in a patient with suspected Guillain–Barrè syndrome: the first case report of an incidental finding in a slit-skin smear

**DOI:** 10.1099/acmi.0.000046

**Published:** 2019-07-26

**Authors:** Soumyabrata Nag, Sneha Gupta, Juhi Sisodia, Richa Misra

**Affiliations:** ^1^ Department of Microbiology, Division of Mycobacteriology, Sanjay Gandhi Postgraduate Institute of Medical Sciences, Lucknow, 226014, India

**Keywords:** Lymphatic filariasis, leprosy, neglected tropical diseases

## Abstract

**Introduction:**

Lymphatic filariasis (LF) and leprosy are both endemic in India. These diseases are on the World Health Organization (WHO) list of neglected tropical diseases (NTDs), as they affect the most marginalized communities in the world, resulting in deformities and functional limitation. We report the first case of asymptomatic filariasis and leprosy co-morbidity in a patient with suspected Guillain–Barré syndrome.

**Case presentation:**

A 55-year-old male who was a farmer by occupation presented to the Neurology Outpatient Department (OPD) of our institute with complaints of weakness in all four limbs for the last 15 days. After admission, a detailed history revealed that the patient had been taking multi-drug therapy (MDT) for leprosy for the previous 6 months. After symptomatic management of the presenting complaints, the patient was sent to the Department of Microbiology for a consultation and six-site slit-skin sampling. The initial screening of Ziehl–Neelsen (ZN)-stained smears under a 10× objective led to the incidental finding of sheathed structures resembling microfilaria (Mf) on the smear made from ear lobules. In addition, short acid-fast bacilli (AFB) were also observed under the oil-immersion objective.

**Conclusion:**

We emphasize that a high index of suspicion and thorough screening of smears by a microbiologist is essential in specimens obtained from any body site.

## Introduction

Lymphatic filariasis (LF) and leprosy are both endemic in India. These diseases are on the World Health Organization (WHO) list of neglected tropical diseases (NTDs) as they affect the most marginalized communities in the world, resulting in deformities and functional limitation [1, 2]. The WHO launched its Global Programme to Eliminate Lymphatic Filariasis (GPELF) in 2000 with the goal of eliminating the disease as a public health problem by 2020 [3]. In 2016, the WHO also launched the Global Leprosy Strategy 2016–2020, with the aim of improving health care coverage and reducing the deformities associated with leprosy [4]. We report the first case of asymptomatic filariasis and leprosy co-morbidity in a patient with suspected Guillain–Barré syndrome (GBS).

LF, commonly known as elephantiasis, is the second most common vector-borne parasitic disease after malaria [1]. One-third of people infected with LF live in India. LF is caused by thread-like worms (nematodes) that inhabit the lymphatic vessels and lymph nodes of humans. Three species of filarial worms, *Wuchereria bancrofti*, *Brugia malayi* and *Brugia timori* cause LF. Microfilariae (Mf), the larval form, are transmitted during a blood meal by infected mosquitoes that serve as the intermediate host. A wide range of mosquitoes can transmit the parasite, depending on the geographical area. The most common vector in Africa is *Anopheles*, while in the Americas it is *Culex*. *Aedes* and *Mansonia* can also transmit the infection in the Pacific and in Asia. During a blood meal, an infected mosquito injects third-stage filarial larvae into the skin of a human host. The larvae develop into adult worms that reside in the lymphatics and produce Mf, which are responsible for transmission of the disease. The Mf migrate into the lymph and enter the bloodstream, reaching the peripheral circulation. A mosquito ingests Mf during a blood meal. After ingestion, they lose their sheaths and penetrate the mid-gut of the mosquito to reach the thoracic muscles. There, they develop into first stage larvae and subsequently into third-stage larvae, which migrate to the proboscis and can transmit infection to another human when the mosquito takes a blood meal [5, 6].

Leprosy is a chronic infectious disease caused by *Mycobacterium leprae *, an acid-fast rod-shaped bacillus. Transmission of the disease is probably by inhalation of droplets containing the causative agent, although contact transmission has also been proposed [7]. The incubation period can be as long as 20 years and host immunity plays an important role in disease progression and control [8]. India contributes almost 95 % of the world’s cases of leprosy each year and accounts for 60 % of new leprosy cases reported globally. This translates to a prevalence of 0.66 per 1 00 000 people. In India, indigenous communities known as Adivasis bear the major burden of disease. They accounted for 13·3 % of new cases of leprosy in 2009, while in 2017 that proportion rose to 18·8 % [9, 10]**.**

Leprosy is classified as paucibacillary (PB) or multibacillary (MB), based on the number of skin lesions, the presence of nerve involvement and microscopic observation of acid-fast bacilli (AFB). A definite diagnosis is made when at least one of the following three cardinal signs are present: (i) definite loss of sensation in a pale (hypo-pigmented) or reddish skin patch; (ii) thickened or enlarged peripheral nerves with loss of sensation and/or weakness of the muscles supplied by that nerve; or (iii) the presence of AFB in a slit-skin smear [8]. The Ziehl–Neelsen method for acid-fast staining here uses 5 % sulphuric acid as a decolourizing agent [7]. Tissue biopsies of affected sites in cases of MB leprosy may reveal typical histopathological changes that show large numbers of foam cells. However, as per the guidelines issued by the Government of India, bacteriological examination is not mandatory to start treatment for leprosy [11].

## Case report

**Fig. 1.**
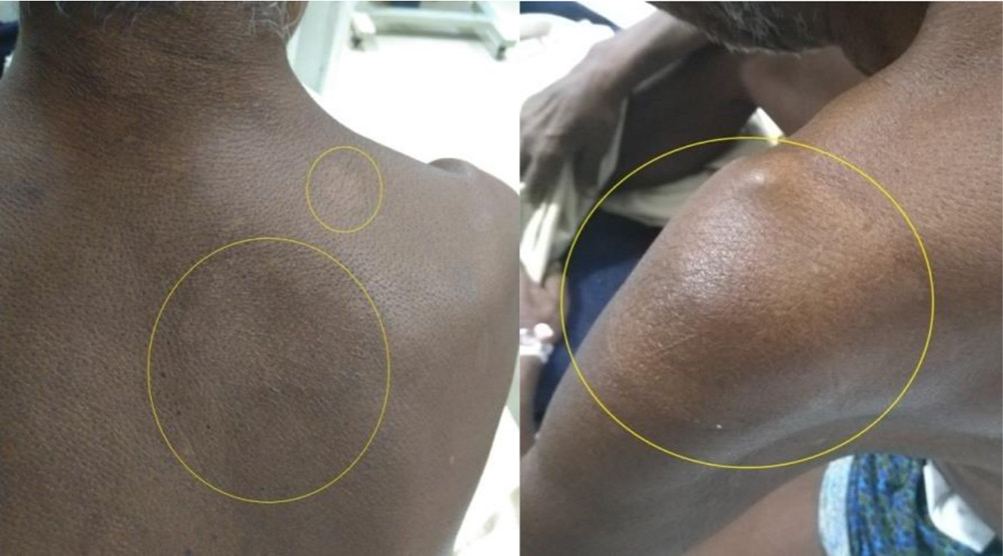
Hypopigmented and anaesthetic patches (yellow circles) on skin over right scapula and left deltoid region.

**Fig. 2.**
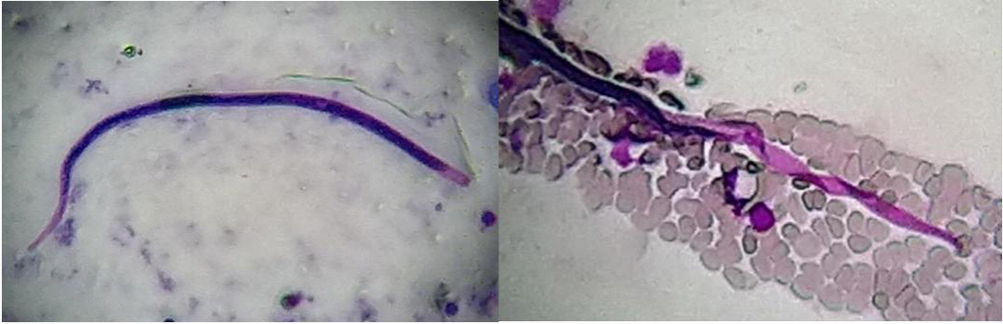
Sheathed Mf observed in slit-skin smear from ear lobule stained using the Ziehl–Neelsen method with 5 % sulphuric acid as a decolourizer (magnification, 10×).

**Fig. 3.**
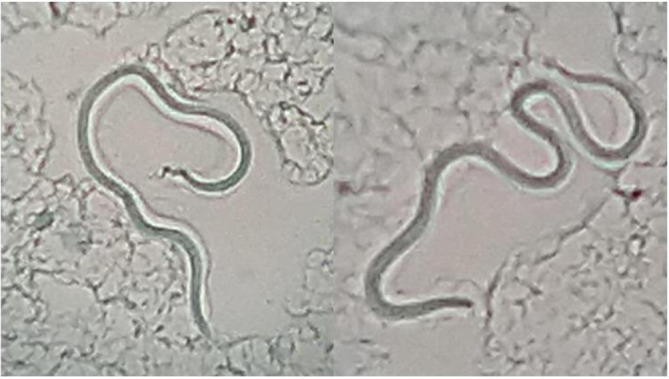
Mf observed in Giemsa-stained peripheral blood smear. (Magnification, 10×). Note the absence of nuclei at the tail tip.

A 55-year-old male who was a farmer by occupation presented to the Neurology Outpatient Department (OPD) at the Sanjay Gandhi Postgraduate Institute of Medical Sciences, Lucknow, India, in September 2018, with complaints of weakness in all four limbs for the last 15 days. About 1 week prior to the onset of weakness, the patient gave a history of fever with flu-like symptoms that subsided on taking antipyretics. The patient also gave a history of a tingling sensation in the toes of both feet and the fingers of the left hand, followed by gradual weakness in all four limbs at the same time. A provisional diagnosis of GBS was made and the patient was admitted to the Neurology ward. After admission, a detailed history revealed that the patient was a diagnosed case of leprosy for which he had been taking multi-drug therapy (MDT) for the previous 6 months. He had noticed hypo-pigmented patches with loss of sensation on the left forearm about 8 months previously, for which he had been prescribed rifampicin, dapsone and clofazimine by a physician in his home town (Fig. 1). After symptomatic management of the presenting complaints, the patient was sent to the Department of Microbiology for a consultation and six-site slit-skin sampling. The following six sites were sampled for microscopy for *M. leprae *: skin lesions, earlobes, eyebrows, elbow, chin and back. The selected skin site was first cleaned with 70 % alcohol. The skin was then pinched up and raised between the thumb and index finger of the left hand to squeeze out blood from the body part and minimize bleeding. With the point of a sterile scalpel blade a 5 mm long and 2–3 mm deep incision was made to create a slit. The bottom and the sides of the slit were scraped to obtain sufficient material, which was then transferred to a clean new glass slide to obtain a uniform and thin smear with an average diameter of 5–7 mm [12]. The smear was air-dried and fixed by passing the slide over the flame. Ziehl–Neelsen staining was performed for direct microscopic examination using 5 % sulphuric acid for decolourization [7]. Initial screening of the slides under a 10× objective led to the incidental finding of sheathed structures resembling Mf on the smear made from ear lobules (Fig. 2). In addition, short AFB were observed under the oil-immersion objective (100×). A tentative diagnosis of co-infection with asymptomatic filariasis and leprosy was made. After the patient was admitted, both thick and thin blood smears were made from blood samples collected at 10 pm. Microscopic examination of Giemsa-stained smears showed multiple sheathed Mf with an absence of nuclei at the tail tip (Fig. 3). A diagnosis of asymptomatic filariasis caused by *W. bancrofti* was made and the patient was initiated on diethylcarbamazine (DEC) at a dose of 6 mg/kg/day in divided doses.

## Discussion

GBS is a rare, rapidly progressive autoimmune polyradiculoneuropathy that may be preceded by respiratory tract or gastrointestinal infection [13]. It can also be a sequelae of a number of diseases, including leprosy and filariasis. In our case, the exact precipitating factor for GBS could not be ascertained for sure. The patient had been treated for leprosy for the previous 6 months without any symptoms of LF. Because his weakness started 1 week after a brief episode of fever with flu-like symptoms, we assumed that the respiratory symptoms were the precipitating cause of GBS.

We report the first case of ‘asymptomatic’ filariasis and leprosy co-morbidity in a patient with suspected GBS. In our patient, in addition to the AFB observed in the slit-skin smear made from ear lobules, sheathed structures resembling Mf were also seen as an incidental finding. We have only come across two cases of patients with LF and leprosy co-morbidity in the literature. Both cases were symptomatic and the patients suffered from grade 2 disability as well as chronic lymphedema in the same limb [14].

Our patient suffered from grade 1 disability, i.e. impaired sensation but no visible impairments. The WHO has suggested a ‘disability classification’ in leprosy patients since 1960 [15]. However, this classification has been revised twice, as a four-point scale in 1970 and a three-point scale in 1988. The main objectives of disability grading are to assess the burden of impairment attributable to leprosy in the community so as to plan necessary actions, to use it as an indicator for assessing the performance of elimination programmes and to grade the potential for preventing disabilities in individual patients. More recently, the individual impairment grades for eyes, hands and feet are summed to compute an ‘eye, hands, feet’ (EHF) score. The EHF sum score is obtained by adding the maximum grade for each of six body sites (eyes, hands and feet), and it can range from 0 to 12[15].

The bacterial load in leprosy can be ascertained by the bacteriological index (BI), which is calculated by counting bacilli in six to eight stained smears under the oil-immersion field. The morphological index (MI) is calculated by counting the numbers of solid-staining acid-fast rods. Only the solid-staining bacilli are considered to be viable. It is a prognostic marker for disease relapse and/or drug resistance [16].

Current guidelines for the treatment of leprosy recommend a three-drug regimen of rifampicin, dapsone and clofazimine for all patients, with 6 months of treatment for PB disease and 12 months for MB leprosy [8]. The advantages are that in endemic areas the same blister pack can be used for treating both types of disease, in addition to decreased impact of misclassification of MB leprosy as PB leprosy, since all patients will receive a three-drug regimen. Early diagnosis and complete treatment with MDT remain the key strategies for reducing the disease burden of leprosy [8, 9].

Filariasis remains subclinical in approximately two-thirds of infected individuals. The remaining one-third suffer from chronic manifestations, such as lymphedema, elephantiasis and hydrocele [17, 18]. According to the WHO, LF is the second most common cause of long-term disability, after mental illness [1]. The standard method for the diagnosis of filariasis is to find Mf in Giemsa-stained thick or thin peripheral blood smears [18]. Nathan and Raccurt have reported higher concentrations of Mf in capillary blood from the earlobe in infections caused by *Mansonella ozzardi* [19]. Species identification may be performed using other stains, such as Wright’s, Papanicolaou’s or Delafield’s haematoxylin, which differentiate the morphological features of the parasite [18]. Characteristically, in *W. bancrofti* the column of nuclei does not extend to the end of the tail, while the widely separated sub-terminal and terminal compact nuclei in the tail are the key diagnostic features in *B. malayi* (Table 1). For increased detection sensitivity in microscopy, Knott’s centrifugation technique may be used for preparing smears [20]. The Mf of *W. bancrofti* and *Brugia* species exhibit nocturnal periodicity and an accurate diagnosis is best achieved on smears collected between 10 pm–2 am. A rapid immunochromatographic test (ICT) for the detection of *W. bancrofti* antigen is being used widely in LF elimination programmes in endemic areas. With this test, Mf can be detected within 10 min independent of the periodicity [21].

**Table 1.** Characteristics of different Mf causing human infection

**Species**	**Sheath**	**Length (µm**)	**Width (µm**)	**Tail**	**Key features of microfilaria**
*Wuchereria bancrofti*	Present	244–296	7.5–10.0	Tapered, anucleate	Short head space, dispersed nuclei, sheath unstained in Giemsa, body in smooth curves
*Brugia malayi*	Present	177–230	5.0–6.0	Tapered, subterminal and terminal nuclei widely separated	Long head space, sheath stains pink in Giemsa
*Brugia timori*	Present	265–323	4.4–6.8	Tapered, subterminal and terminal nuclei widely separated	Long head space, sheath unstained in Giemsa
*Loa loa*	Present	231–250	5.0–7.0	Tapered; nuclei irregularly spaced to end of tail	Sheath unstained in Giemsa
*Mansonella ozzardi*	Absent	163–203	3.0–5.0	Long, slender pointed; anucleate	Small size; long slender tail; aperiodic
*Mansonella perstans*	Absent	190–200	4.0–5.0	Bluntly rounded, nuclei to end of tail	Small size; aperiodic
*Mansonella streptocerca*	Absent	180–240	5.0–6.0	Bluntly rounded, bent into hook; nuclei to end of tail	Slender shape; occurs in skin
*Onchocerca volvulus*	Absent	304–315	5.0–9.0	Typically flexed; tapered to a point; anucleate	Occurs in skin

Mass drug administration programmes (MDAs) with diethylcarbamazine and albendazole, or albendazole and ivermectin, conducted for 4 to 6 years, to interrupt the transmission of the parasite is the main strategy for eliminating the disease [21–24]. As per the WHO guidelines, any area (usually an administrative unit) in an endemic country with an Mf prevalence of 1 % or more is to be covered under the MDA programme [25, 26].

A review of the literature revealed several published case reports in which Mf were detected as a coincidental finding in several unusual sites, such as a thyroid aspirate, bronchial washings, cervico-vaginal smear, various benign and malignant tumours, hydrocele fluid, etc. [27]. However, our case is the first in which Mf have been observed along with AFB in a slit-skin smear from ear lobules in a leprosy patient without any symptoms of LF.

## Conclusion

In conclusion, we emphasize that a high index of suspicion and thorough screening of smears by a microbiologist is essential in specimens obtained from any body site. Mf were not observed in a peripheral blood smear examination after 5 days of therapy with DEC. The patient was discharged after power improved in his limbs (upper limbs 5/5 and lower limbs 4/5). At the time of the submission of this report, the patient is doing well. When contacted by telephone, he informed us that there was no weakness in his limbs and he could perform his daily tasks. He was still continuing his medications for leprosy.
